# Temporal Relationship of Ocular and Tail Segmental Movements Underlying Locomotor-Induced Gaze Stabilization During Undulatory Swimming in Larval Xenopus

**DOI:** 10.3389/fncir.2018.00095

**Published:** 2018-10-29

**Authors:** Julien Bacqué-Cazenave, Gilles Courtand, Mathieu Beraneck, François M. Lambert, Denis Combes

**Affiliations:** ^1^CNRS UMR 5287, Institut de Neurosciences Cognitives et Intégratives d’Aquitaine, Université de Bordeaux, Bordeaux, France; ^2^CNRS UMR 8119, Center for Neurophysics, Physiology, and Pathology, Université Paris Descartes, Paris, France

**Keywords:** oculomotor, efference copy, locomotion, tail undulation, gaze, xenopus

## Abstract

In larval xenopus, locomotor-induced oculomotor behavior produces gaze-stabilizing eye movements to counteract the disruptive effects of tail undulation during swimming. While neuronal circuitries responsible for feed-forward intrinsic spino-extraocular signaling have recently been described, the resulting oculomotor behavior remains poorly understood. Conveying locomotor CPG efference copy, the spino-extraocular motor command coordinates the multi-segmental rostrocaudal spinal rhythmic activity with the extraocular motor activity. By recording sequences of xenopus tadpole free swimming, we quantified the temporal calibration of conjugate eye movements originating from spino-extraocular motor coupled activity during pre-metamorphic tail-based undulatory swimming. Our results show that eye movements are produced only during robust propulsive forward swimming activity and increase with the amplitude of tail movements. The use of larval isolated *in vitro* and semi-intact fixed head preparations revealed that spinal locomotor networks driving the rostral portion of the tail set the precise timing of the spino-extraocular motor coupling by adjusting the phase relationship between spinal segment and extraocular rhythmic activity with the swimming frequency. The resulting spinal-evoked oculomotor behavior produced conjugated eye movements that were in phase opposition with the mid-caudal part of the tail. This time adjustment is independent of locomotor activity in the more caudal spinal parts of the tail. Altogether our findings demonstrate that locomotor feed-forward spino-extraocular signaling produce conjugate eye movements that compensate specifically the undulation of the mid-caudal tail during active swimming. Finally, this study constitutes the first extensive behavioral quantification of spino-extraocular motor coupling, which sets the basis for understanding the mechanisms of locomotor-induced oculomotor behavior in larval frog.

## Introduction

During locomotion, compensatory eye movements are produced to offset head/body movements, hence ensuring stable vision. These gaze stabilizing reflexes were classically attributed to visual and vestibular-dependent pathways (Raphan et al., 1979; Paige, 1983; Straka and Dieringer, 2004). However, over the last decade evidence obtained from *in vitro* experiments on *Xenopus laevis* revealed that during swimming, gaze stabilization depends on feedforward signaling directly from the spinal locomotor central pattern generator (CPG) (Combes et al., 2008; Lambert et al., 2012; von Uckermann et al., 2013, 2016). In pre-metamorphic animals, this CPGs efference copy provokes alternating bursts in extraocular motor nerves innervating synergistic pairs of lateral and medial rectus (LR, MR) muscles. In consequence, horizontal body movements consecutive to animal motion are compensated by horizontal left/right eye movements in the opposite direction. So far, studies in larval Xenopus were focused on the description of central pathways supporting such a locomotor-induced gaze stabilizing mechanism (Lambert et al., 2012). In particular, we demonstrated that the spinal drive underlying extraocular compensatory movements depends on fictive swimming activity produced by the 10 first spinal segments. However, the swimming behavior can’t be explained with a restricted left-right alternating tail beat, but rather depends on a complex kinematic resulting from the sinewave-like undulation of the tail (Wassersug and von Seckendorf Hoff, 1985). Such undulatory movement is produced by a rostro-caudal sequential activation of multi-segmental spinal CPGs responsible for adjacent myotome contraction (Combes et al., 2004). The delay between intersegmental spinal CPGs’ activities determines the temporal parameters of the undulatory swimming and the temporal coordination between the different tail segments. Therefore, the spino-extraocular motor command has to transform a multi-segmentally propagated CPG rhythmic pattern in a binary left-right alternating bursting discharge in synergistic LR/MR motor nerves. This raises the question as to which component of the tail movement is the locomotor efference copy temporally set, generating appropriate eye movements and compensating undulatory swimming?

To address this question, we used intact animals as well as semi-intact preparations at pre-metamorphic larval stages to record tail and eye movements, or isolated *in vitro* preparation to record the corresponding spinal and extraocular motor nerve activity during spontaneous swimming episodes, either in natural or fictive swimming experimental conditions. Our results demonstrate that the locomotor-induced oculomotor behavior, elicited by an internal efference copy signaling from rostral spinal motor networks, is specifically tuned to produce conjugate eye movements that compensate the mid-caudal tail undulatory pattern during propulsive swimming.

## Materials and Methods

### Animals

Experiments were conducted on the South African clawed toad *X. laevis* obtained from the Xenopus Biology Resources Centre in France (University of Rennes 1^1^). Animals were maintained at 20–22 °C in filtered water aquaria with a 12:12 h light/dark cycle. Developmental stages were sorted according to external body criteria (Nieuwkoop and Faber, 1956), and experiments were performed on larvae from stage 53 to 57. All procedures were carried out in accordance with, and approved by, the local ethics committee (#2016011518042273 APAFIS #3612).

### Video Recordings of Tail Undulation During Free Swimming

**FIGURE 1:**
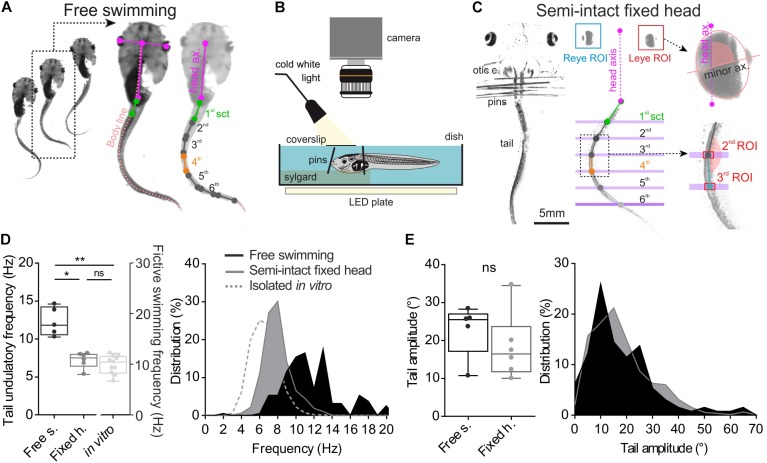
Method and comparison of swimming behavior in two different experimental conditions. **(A)** Free tadpole swimming was recorded by a camera placed above the tank. Then homemade imageJ macro was used to track eye position frame by frame and create body skeleton (light pink dot line). This macro provided XY coordinates of each point (color dots), used to calculate right/left angular excursion of the first tail section (1st sct, green line) and the body axis (body ax., pink line), and between each tail section. **(B,C)** Semi-intact preparations used to quantify the oculomotor behavior induced by swimming. To avoid any visuo-vestibular sensory inputs, optic cranial nerves were cut and the tadpole’s head was fixed on the Sylgard by pins closed to its otic capsules (otic c.). The tail was free to swim in a deeper compartment. Homemade software tracked lateral movements and measured lateral angle values for each eye (eye ROI) between eye axis (minor ax.) and head axis (head ax.). Coordinates of each tail section (green, gray and blue dot) were calculated from tail ROI. **(D)** Mean undulation frequency of the first tail section during swimming was significantly lower (Dunn’s multiple comparisons test, *p* ≤ 0.05) in head fixed (black bar, Fixed h., *n* = 7) preparations than in free swimming condition (dark gray bar, Free s., *n* = 5). Mean bursting frequency recorded in spinal rostral ventral root (light gray bar, *in vitro*, *n* = 11) was also lower than mean undulatory frequency of the first tail section measured in semi-intact or free swimming animals (Dunn’s multiple comparisons test, *p* < 0.001). **(E)** Averages and range of peak-to-peak tail amplitude movements were similar (Mann–Whitney test, ns) in both experimental conditions.

To study the tail undulation kinematic during free swimming, animals (*n* = 5) were placed in an aquarium (20 × 40 cm) filled with filtered water to a depth of 3 cm. In these behavioral conditions, larvae were able to produce unobstructed swimming sequences up to 20 tail swim cycles. In these experiments, only uninterrupted episodes of swimming with at least 10 consecutive undulatory cycles were analyzed. Video sequences were recorded with a digital camera (Basler, acA1920) positioned above the aquarium. A wide-angle lens (Fujinon 16 mm 1:1.4) was used to monitor tail movements throughout free-swimming episodes. Image acquisition frequencies were of 250 fps. Video sequences were collected on computer through a USB3 interface and stored using the AVI video file format with the Pylonviewer5 software (Basler). Subsequent data analyses were performed using a homemade macro from free Fiji (build on top of the ImageJ2 core, (Schindelin et al., 2012) that measures the angle between adjacent tail sections from a recorded sequence of swimming activity (Figure 1A). Tadpole tracking was performed with the following image process. First, the frame-by-frame position of both eyes in space was detected after image thresholding. Then the user defined the length of reference for the head and the first section of the tail (Figure 1A purple and green segments). Second, based on this reference length, consecutive body sections were modelized from the inter-ocular midpoint segment and the axial linear skeleton obtained from the “morphological skeletonize” ImageJ2 plugin (Figure 1A, pink dashed line named “body line”). Third, frame-by-frame 2D coordinates of each points defining every tail sections were obtained from the displacement of the tadpole body line during the swimming episode (Figure 1A, right image). These XY coordinates were used to calculate the angular excursion of each tail section.

### Video Recordings of Eye and Tail Movement in Fixed Head Semi-Intact Preparations

Semi-intact preparations (Figure 1B) were used to quantify the locomotor-induced oculomotor behavior during undulatory swimming. Larvae were anesthetized in a 0.05% MS-222 solution and placed in oxygenated (95% O_2_, 5% CO_2_) Ringer solution (composition in mM: NaCl, 120; KCl, 2.5; CaCl2, 5; MgCl2, 1; NaHCO3, 15; pH = 7.4). The temperature of bathing solution was controlled and maintained around 18°C. Viscera and telencephalon were removed and animals were fixed dorsal side up to a Sylgard-lined Petri dish. Brainstem was exposed to Ringer solution perfusion. To avoid any visuo-vestibular sensory inputs, the optic nerves were transected bilaterally and the semi-intact preparation was placed in a special chamber where the head was firmly secured to the Sylgard with pins. The tail was free in a deeper compartment to allow unrestrained swimming undulatory movements (Figure 1B). A coverslip was positioned above the head to avoid fluid disturbances due to tail biting during swimming events that would have affected the video-recordings. Swimming related movements of the tail and the eyes were video-recorded at 500 fps with a high speed digital camera (Basler, ac1920) equipped with a micro-inspection lens system (Optem MVZL macro video zoom lens, QIOPTIQ). Automatic tracking of eyes and tail segments was performed using a homemade software coded in Python 3.5 environment. For eye movement measurement, a region of interest (ROI) was first drawn around each eye. Inside each ROI a binary threshold with respect to dark/bright eye areas was used to produce a black/white ellipse-like image of the eye (Figure 1C). The Python software calculated frame by frame the angle between the minor axis of the ellipse and the head axis. For tail movement measurement, virtual line markers were positioned on the image to delimit each tail segment. Each segment ROI was defined by the crossing of the tail with the corresponding line marker (Figure 1C). The software measured frame-by-frame the angle between two consecutive tail segments. The angle of the first segment was calculated relative to the head axis (Figure 1C).

### Extraocular and Spinal Motor Nerve Recordings in Isolated *in vitro* Preparations

Isolated *in vitro* brainstem/spinal cord preparations were used to simultaneously record extracellular activity from extraocular motor nerve innervating the lateral rectus muscle (LR) and spinal ventral roots (Vr) innervating the 5th, 15th, and 20th myotomes (see Lambert et al., 2012; von Uckermann et al., 2016). The dissection began the same way as previously described in the semi-intact preparation section. Then the spinal cord and its Vrs were exposed until segment 22–25 and the rest of the tail were carefully removed. LR motor nerve was disconnected from its target muscle. The preparation was continuously superfused with oxygenated Ringer saline at a rate of 1.5–2.0 ml/min and maintained at 18 ± 0.1°C with a Peltier cooling system. In some preparations, a Vaseline wall was made at the level of the 10–12th spinal segments to perfuse the brainstem and rostral spinal cord (above segment 10) independently from the caudal spinal cord (below segment 10). In this case the caudal spinal cord was superfused with a 10% sucrose solution (in water) to block all neuronal activity in this compartment. Vaseline wall sealing was tested at the end of the experiment by adding fast green colorant in one of the two compartments. Extracellular activities in LR and Vr motor nerves were recorded using adjusted glass suction electrodes connected to a differential AC amplifier (A-M System Model 1700 AC, Carlsborg, United States). Electrophysiological signals were digitized at 10 kHz (CED 1401, Cambridge Electronic Design, United Kingdom), then displayed and stored on a computer for offline analysis.

### Signal Processing and Data Analysis

Raw data from video or electrophysiological recordings were processed off-line and analyzed with Dataview (by W. J. Heitler, University of St Andrews, Scotland).

For video image processing, traces of angular movements from eyes and/or tail sections were first filtered with a 25 Hz low-pass filter. Tail swimming cycles were defined based on angular excursion of the 1st tail section and the null angle excursion of the 1st tail section determined the cycle start. Then maximum angle values of tail sections and eyes (peak sinewave) were detected in each individual swimming cycle. These peak markers were used to calculate instantaneous peak frequency, peak-to-peak latency from the 1st tail section (sct) or eye, and the temporal relationship of consecutive tail sections relative to the 1st tail section or eye.

**FIGURE 2:**
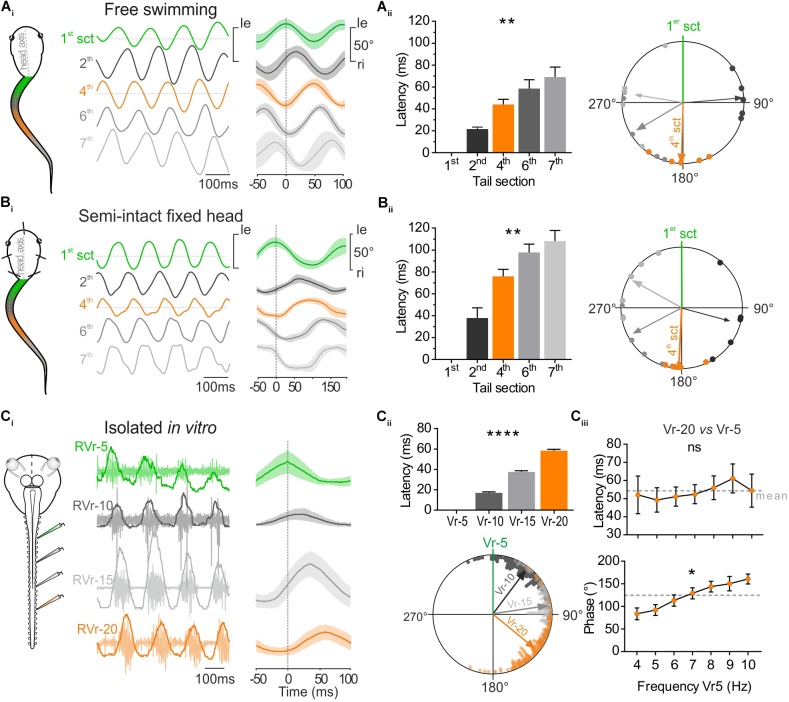
Temporal relationship between tail sections during larval swimming. **(A)** During free swimming **(A_i_)** the sinewave-like undulatory movements of the tail were generated by alternative left and right excursion of 1st tail section (green trace, 1st sct) spread to next caudal sections. Tail sections were video-tracked frame by frame. Traces show angular excursion of each tail sct (in degree) over time. Cycle analysis (25 cycles) shows that the 4th tail section (orange trace, 4th sct) was totally out of phase with the 1st section (1st sct, green trace). **(A_ii_)** Mean latency between oscillations of the 1st and consecutive more caudal tail sections increased gradually and significantly (Kruskal–Wallis test, *p* < 0.01, *n* = 5). Therefore, the phase shift relative to the 1st section (green line) increased significantly (Watson–Williams test, *p* < 0.0001, *n* = 5. The 4th section was significantly out of phase (orange arrow) with the 1st tail section (*V*-test, *p* < 0.001, *n* = 5). **(B)** In semi-intact preparations, overlapping traces (**B_i_**_,_ 50 cycles) showed also that the 4th tail section (orange trace) was in phase opposition with the 1st tail section (green trace). **(B_ii_)** Mean latency between oscillations of the 1st and consecutive more caudal tail sections increased gradually and significantly (Kruskal–Wallis test, *p* < 0.01, *n* = 6). Therefore, the phase shift relative to the 1st section (green line) increased also significantly (Watson–Williams test, *p* < 0.0001, *n* = 5). The 4th section was significantly out of phase (orange arrow) with the 1st tail section (*V*-test, *p* < 0.001, *n* = 5). **(C)** Simultaneous extracellular recordings of spinal ventral roots (Vr) of brainstem-spinal cord preparations isolated *in vitro* showed a rostro-caudal delay typical for fictive swimming. The 20th Vr (orange trace) was consequently totally out of phase with the 5th Vr (green trace, 50 cycles). **(C_ii_)** The delay between the 5th root’s bursts and the next caudal Vrs (10, 15, and 20 successively) increased significantly (Kruskal–Wallis test, *p* < 0.01, 180 cycles) and consequently the phase shift of bursts recorded in Vrs 10, 15, and 20 relative to Vr-5 (green line) also increased significantly (Watson–Williams *F*-test, *p* < 0.0001, 180 cycles). Vr-20 (orange arrow) was significantly in phase opposition with Vr-5 (*V*-test, *p* < 0.0001, 180 cycles). **(C_iii_)** On different *in vitro* preparations, latency and phase relationships of Vr-20 relative to Vr-5 bursts (Vr-20 vs. Vr-5) were calculated for fictive swimming frequencies ranging from 4 to 10 Hz. The latency didn’t change (Kruskal–Wallis test, ns, *n* = 7), whereas the phase relationship increased as the Vr-5 frequency was raised (Watson–Williams *F*-test, *p* < 0.01, *n* = 7).

Electrophysiological recording signal of each motor nerve was rectified about 0 mV and smoothed (moving average method, time constant 25 ms) to obtain an integrated signal. Fictive swimming cycle was determined from the 5th rostral ventral root (Vr-5) bursting discharge. Then an event was generated for each detected peak in Vrs and LR bursts in each fictive swimming cycle. Frequency, latency and temporal relationships were calculated in Dataview. 1st sct or Vr-5 signals were used as the phase marker in phase relationship analysis shown in Figure 2. Leye or LLR signals were used as the phase marker in phase relationship analysis presented in Figures 4, 5. The area under the integrated traces of each burst was measured with the area measurement toolbox in Dataview and enabled evaluation of the strength of each multi-unit burst (inspired from Mulloney, 2005). These area values were used to measure correlation between Vrs and LR bursts.

### Statistics

After signal processing in Dataview, data were analyzed using Prism7 (GraphPad, United States). A normal distribution was verified (D’agostino & Pearson normality test and Shapiro–Wilk normality test for small sample), and thus the results were expressed as the means ± SEM, unless stated otherwise. For swimming parameters (frequency and amplitude) and integrated electrophysiological signals, differences between two results were tested using the unpaired two-tailed Mann–Whitney *U*-test, and Kolmogorov–Smirnov test to compare distribution. To compare several values, non-parametric Kruskal–Wallis test was processed with a Dunn’s multiple comparisons test. Circular data analysis was performed with Oriana 4.02 software (Kovach Computing Services, Wales). The temporal relationships between eye and tail sections angular excursion, or in the Vrs and LR in different preparations were assessed by circular phase analysis of pooled data. The mean vector ‘μ’ and its length ‘r’ indicated the preferred phase and the strength of coupling, respectively. To test if the distribution of mean angle values was non-uniform with a specified mean direction, data was processed with a *V*-test and an expected mean. For non-uniform distributions (tested with the Rayleigh’s uniformity test, *p*), mean phase values of each animal were plotted as the grand mean of the individual means of phase relationship between tail section/Vrs and eye/LR. They were expressed as (μ;r;*p*). Preferred direction of grand mean vector was tested by Moore’s Modified Rayleigh test and the difference on phase relationships between means was evaluated by using the Watson–Williams *F*-test. Linear regression (r^2^) was proceeded to evaluate the kind of correlation between amplitude of angular excursion of eye and tail section, or between burst area of LR and Vrs. Slope (s) of these regression expressed the gain between the two factors tested. Regression results were expressed as (r^2^, s). Differences between results values were taken to be significantly different at *p* < 0.05 (^∗^*p* < 0.05; ^∗∗^*p* < 0.01; ^∗∗∗^*p* < 0.001; ^∗∗∗∗^*p* < 0.0001; ns: non-significant). All mean numerical values are given in a Supplementary Data Source.

## Results

Before analyzing tail and eye movements coordination, a fine description of the sinewave undulation of the tail (Wassersug and von Seckendorf Hoff, 1985) as well as the relationships between this swimming behavior and the activity underlying segmental CPGs (Combes et al., 2004) was necessary. Therefore, we first quantified during undulatory swimming the delay between the movement of the main sections of the tail and the CPGs activity of the associated spinal motor local networks in our three experimental conditions: free swimming, head-fixed and *in vitro* preparation.

### Quantitative Description of Tail Movements During Larval Swimming

Video analysis of free swimming sequences (Figure 1A) obtained from 5 larvae showed a mean tail swimming frequency of 12.26 ± 0.84 Hz with a mean tail amplitude of 22.77 ± 3.09° (Figures 1D,E). The tail undulatory frequencies were mainly distributed between 6 and 20 Hz (Wassersug and von Seckendorf Hoff, 1985; von Seckendorff Hoff and Wassersug, 1986) with the majority of them ranging from 9.52 to 13.33 Hz (25th and 75th percentile, respectively, Figure 1D, dark box and area). The tail amplitudes stretched from 5° to 80° with 70% comprised between 10° and 30° (Figure 1E, dark box and area). In semi-intact fixed head condition (Figure 1B) the tail frequency ranged from 4 to 14 Hz (mean frequency: 7.20 ± 0.42 Hz) with 90% comprised between 6 and 10 Hz (Figure 1D, gray box and area). The distribution of the tail amplitudes in semi-intact condition was comparable (Mann–Whitney test, ns) to that observed during the free swimming condition with a mean amplitude of 18.32 ± 3.55° (Figure 1E). Finally, during fictive swimming events recorded in isolated *in vitro* brainstem-spinal cords, the mean bursting frequency measured from spinal ventral roots was lower (6.60 ± 0.36 Hz; Figure 1D) than frequencies measured from undulation of tail sections during real swimming sequences. The distribution of the frequencies in fictive swimming sequences was mainly comprised between 4 and 10 Hz (Figure 1D, gray dotted line).

### Rostro-Caudal Timing of Segmental Tail Movement and Its Spinal Motor Command

The undulatory movement of the tail was video-tracked to measure frame-by-frame the angular excursion of the different tail sections (Figure 1C). The temporal parameters of those tail movements were compared in free swimming (Figure 2A_i_) and head-fixed larval preparations (Figure 2B_i_). The mean rostro-caudal latency between the movement of the first and adjacent consecutive tail sections increased in both conditions (Figures 2A_ii_,B_ii_, left histogram) resulting in an increasing phase shift between the more caudal tail sections (Figures 2A_ii_,B_ii_, right polar plot). As a consequence, the mid-caudal section of the tail (4th section) was undulating in complete phase opposition with the most rostral section of the tail (1st section) with a phase lag of 181.51 ± 13.64° in free swimming and 182.76 ± 6.74° in head-fixed animals (Figures 2A_ii_,B_ii_; Wassersug and von Seckendorf Hoff, 1985). The mean latencies relative to the 4^th^ section of the tail were lower (Sidak’s multiple comparisons test, *p* < 0.01) in free swimming compared to head-fixed preparations (Figure 2A_ii_,B_ii_) due to the higher swimming frequency expressed in free swimming (Figure 1D). In contrast phase relationships between free swimming and head fixed conditions did not change significantly (Watson–Williams test, ns). In isolated *in vitro* preparations, locomotor bursting activity was recorded from ventral roots (Vr) of several spinal segments during fictive swimming sequences (Figure 2C_i_). The temporal pattern of locomotor bursts recorded from Vr-5, 10, 15, and 20 was comparable to the rostro-caudal pattern observed between tail segments in free swimming and head-fixed conditions. The increasing rostro-caudal delay between the 5th and the following caudal Vr bursts induced an increasing phase shift as the bursts propagated caudally (Figure 2C_ii_) corresponding to fictive forward swimming. In the example shown in Figure 2Ci, the latency measured between the 5th and the 20th Vr locomotor burst was 58.41 ± 1.27 ms with a phase shift of 129.18 ± 2.28°for fictive swimming frequencies ranging from 7 to 9 Hz.

Spontaneous fictive swimming recorded from isolated CNS sequences can occur in different frequency ranges. We therefore evaluated the temporal parameters of burst propagation along the spinal cord according to the swimming frequency. The mean latency between Vr-5 and Vr-20 in all preparations was of ∼58 ms (Figure 2C_iii_) and did not show a significant change in the range of the observed swimming frequencies (4–10 Hz). Thus, the phase shift between successive Vrs from Vr-5 to Vr-20 increased significantly with increasing fictive swimming frequencies.

Altogether these results demonstrate that head fixed semi-intact preparations and isolated *in vitro* preparations exhibit swimming activity with a temporal organization highly comparable to *in vivo* free swimming behavior. Consequently, this undulatory pattern generates a rostro-caudal phase shift for which the mid-caudal part of the tail (17–22th myotome segments) oscillates in phase opposition with the rostral part of the tail (1–7th myotome segments). As the locomotor CPG rhythmic activity exhibits a constant latency between rostro-caudal segments, the temporal relationship between rostral and mid-caudal tails directly depends on the swimming frequency.

### Locomotor-Induced Eye Movements Result From Propulsive Swimming Behavior

**FIGURE 3:**
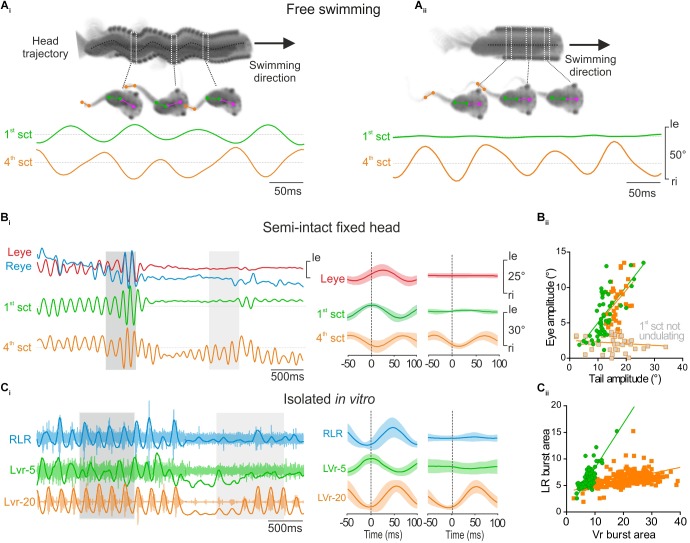
Propulsive swimming behavior triggered locomotor-induced eye movement. **(A)** Tadpole swimming (freely behaving) captured on camera during strong propulsive swimming which provoked angular head movements (head trajectory, dark dot line) **(A_i_)** or during weak swimming without oscillatory head movements **(A_ii_)**. During strong propulsion **(Ai)**, the 1st tail section (green trace, 1st sct) undulated in phase opposition with the 4th tail section (orange trace, 4th sct). In contrast, during slow swimming **(A_ii_)**, the more rostral (1st) tail section showed no oscillation. **(B)** When the 1st tail section (green trace) undulated in head fixed condition (dark gray area), related to strong swimming, angular excursions of the 1st tail section were always propagated to 4th tail section, and appeared also associated with conjugated eye movements (red and blue traces, respectively, left and right eyes) with a weak shift phase (see left overlapping traces, 50 cycles). In contrast, locomotor-induced eye movements didn’t occur when the 4th tail section undulated alone (see light gray area and right overlapping traces, 50 cycles). **(B_ii_)** The magnitude of lateral eye angular excursion was linearly correlated with the amplitude of the 1st tail section undulatory movements with an average gain close to 0.5 (green circle and line, *r*^2^ = 0.46; *s* = 0.51 ± 0.08) during strong propulsive swimming. Therefore, the correlation between the amplitude of eye angular excursion and the amplitude of the 4th tail section movements was also linear with a gain highly similar (orange square and line, *r*^2^ = 0.21; *s* = 0.47 ± 0.16) in the same condition. In contrast, the amplitude of eye movements was not correlated with the amplitude of 4th tail section movements when tadpole generated slow swimming (gray square, *r*^2^ = 0.02; *s* = –0.02 ± 0.02). **(C)** Simultaneous recordings of the right lateral rectus (RLR), the 5th contralateral spinal ventral root (LVr-5) and the 20th left spinal ventral root (LVr-20) activities from an isolated *in vitro* preparation **(C_i_)**, during a strong (dark gray area) or weak (light gray area) swimming. Integrated traces (dark lines) were superimposed on raw traces (light traces). Bursting activities recorded on RLR (blue trace) occurred in phase with LVr-20 (orange trace) and in phase opposition with LVr-5 (green trace) (see mean overlapped traces on left, 120 cycles). **(C_ii_)** Burst areas of LR were strongly linearly correlated (*r*^2^ = 0.80; *s* = 0.83 ± 0.04) with Vr-5 burst area during propulsive swimming (green circle and line). Conversely during weak swimming, only LVr-20 discharged (mean overlapped traces on right, Ci, 41 cycles) and thus LR burst areas were weakly correlated with the Vr-20 bursts area (orange square and line, *r*^2^ = 0.18; *s* = 0.11 ± 0.01).

Larval body kinematic analysis performed at 500 fps revealed two different tail movement patterns directly related to the swimming behavior and the involvement or not of rostral tail segments. Efficient propulsive swimming behavior was characterized by a strong bilateral excursion of the rostral part of the tail together with a pronounced angular movement of the head (Figure 3A_i_, 1st section; Hanzi and Straka, 2017). Inversely, slow swimming behavior was characterized by an absence of undulation of the rostral part of the tail and a forward head translation despite a robust angular excursion of more caudal tail sections (Figure 3A_ii_). Therefore, the undulation amplitude of the first section of the tail enabled us to discriminate these two characteristic swimming behaviors in head-fixed semi-intact preparations, and thus to identify the eye movement produced in relation to tail movements (Figure 3B). In head-fixed preparations the intensity of tail movements fluctuated constantly recruiting more or less caudal sections of the tail (Figure 3B_i_). In this condition, the locomotor-induced oculomotor behavior was clearly correlated to undulatory movements of the rostral tail section (Figure 3B_i_, left panel). The smallest detectable swimming-driven eye movements (∼4°) were recorded for a 1st tail section excursion of ∼10 ° (Figure 3B_ii_). Robust conjugate eyes movements (>5°) were produced for angular excursion of the 1st tail section higher than 10° and increased linearly up to ∼20°(Figure 3B_ii_, green circles). No conjugate eye movement was recorded when the rostral tail did not produce significant undulatory movements (Figure 3B_i_, right panel), despite high angular excursion of mid-caudal tail section (Figure 3B_i_, right panel, 4th section and B_ii_, gray squares). Similar to the *in vivo* swimming behavior, the recruitment of more or less rostro-caudal spinal segments fluctuated in isolated *in vitro* preparations (Figure 3C_i_). Both rostral (Vr-5) and caudal VRs (Vr-20) were rhythmically active during robust fictive swimming sequences (Figure 3C_i_, left panel), corresponding to the CPG motor pattern produced for propulsive swimming behavior. In contrast only caudal Vrs were rhythmically active during weak fictive swimming periods (Figure 3C_i_, right panel), corresponding to a motor pattern that induces slow swimming behavior. Robust extraocular motor discharge was recorded in the lateral rectus (LR) motor nerve only when the rostral spinal Vr-5 displayed a rhythmic activity (Figure 3C_i_, left panel), with a linear relationship between activities recorded in the two nerves (ratio closed to 0.5, Figure 3C_ii_, green circles). Conversely, no activity was observed in the LR motor nerve when the rostral spinal Vr-5 was silent even if the caudal Vr-20 exhibited locomotor rhythmic discharge (Figure 3C_i_, right panel). Consequently, the relationship between LR and mid-caudal Vrs activities was poor (Figure 3C_ii_, orange square). These results show that locomotor-induced oculomotor behavior is produced by a spino-extraocular motor command originating from rostral CPGs like originally described by Lambert et al., 2012, but clarify that this occurs only for propulsive swimming behavior in larval frog.

### Locomotor-Induced Conjugate Eye Movements Compensate Mid-Caudal Tail Undulation

**FIGURE 4:**
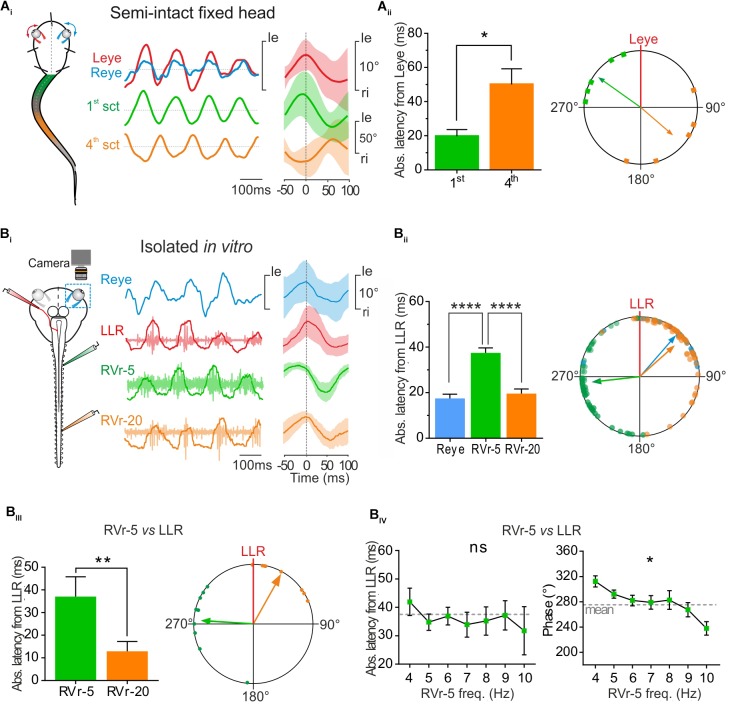
Conjugate eye movements generated by propulsive swimming compensate mid-caudal tail undulation. **(A)** In a head fixed preparation, the left and right eyes (respectively, red, and blue traces) rotated simultaneously in phase with undulation of the 1st tail section and in phase opposition with the 4th tail section. Latency between the peak of eye movements and the 1st tail section (green bar) was significantly lower (Mann–Whitney test, *p* < 0.05, *n* = 5) than latency between movements of the eyes and the 4th section of the tail (orange bar), resulting in a significant difference in phase relationship between the 1st and the 4th tail section relative to eye movement (polar plot). **(B)** Simultaneous recordings of right eye (Reye, blue trace) movements and activity in the left lateral rectus (LLR, red trace) nerve and the 5th (RVr-5) and 20th (RVr-20) right spinal ventral root during *in vitro* fictive swimming. **(B_i_)** Real (blue) and fictive (red) leftward excursions of the eyes were in phase lag with bursting discharge in the 5th Vr and in reduced phase lead with the 20th Vr, compatible with a compensatory eyes movement during mid-caudal tail undulation (Vr-20). **(B_ii_)** Absolute average latency between the Reye movement (blue bar) or RVr-20 bursts (orange bar) and LLR firing were comparable, but significantly lower (Dunn’s multiple comparisons test, *p* < 0.0001, 61 cycles) than average latency between RVr-5 and LLR bursting (green bar). A significant difference is observed between the phase lead of the 5th ventral root bursting relative to that of the LLR and the phase lag of Reye movement and RVr-20 bursting relative to LLR firing (Watson–Williams *F*-test, *p* < 0.001, 61 cycles). **(B_iii_)** On different *in vitro* preparations, the absolute mean latency between RVr-20 and LLR was weak (12.93 ± 4.31 ms) and significantly lower (Mann–Whitney test, *p* < 0.01, *n* = 8) than the absolute mean latency between RVr-5 and LLR (37.11 ± 2.74 ms). Therefore, bursting discharges of LLR and RVr-20 (orange arrow) were nearly in phase (37.92°, 0.689, *p* < 0.001) while bursting discharges of RVr-5 (green arrow) were in phase lead with the LLR (274.41°, 0.65, *p* < 0.001). These temporal relationships were significantly different (Watson–Williams *F*-test, *p* < 0.0001, *n* = 8). **(B_iv_)** The latency between LLR and RVr-5 bursts (left curve) remained constant for fictive swimming frequencies ranging from 4 to 10 Hz (Kruskal–Wallis test, ns, *n* = 8). According to those latency results, phase shift decreased significantly with increasing fictive swimming frequency (Watson–Williams *F*-test, *p* < 0.05, *n* = 8).

During propulsive swimming behavior rostral and mid-caudal tail sections were coordinated with a frequency-dependent phase opposition (Figure 2). In addition rostral CPGs, responsible for movement of the 1st tail section, generated a spino-extraocular motor command eliciting locomotor-induced conjugate eye movements (Figure 3). Head-fixed semi-intact preparations produced characteristic conjugate eye movements in phase synchrony with the first section of the tail and in phase opposition with the 4th section of the tail during propulsive swimming sequences (Figure 4A_i_). Quantitative analysis revealed that the eyes moved with an absolute mean peak-to-peak latency of 50.14 ± 9.15 ms with the 4th tail section and 19.71 ± 3.98 ms with the 1st tail section (Figure 4A_ii_, histogram; Supplementary Figure S1), generating a mean phase lead of 130.70 ± 28.05° with the 4th tail section and a mean phase lag with the 1st tail section of 305.25 ± 15.98°(Figure 4A_ii_, polar plot; Supplementary Figure S1).

To correlate locomotor-induced eye movements with their spino-extraocular motor substrate, two isolated *in vitro* preparations were performed with one eye kept intact whereas the other eye was dissected out to record the LR motor nerve (Figure 4B_i_, left scheme). Robust fictive swimming episodes elicited typical locomotor rhythmic activity (Figure 4B_i_) with an important phase shift between rostral and mid-caudal ventral roots (Vr-5 vs. Vr-20, see Figure 4B_i_, right side cycle average) as previously described in Figure 2. This fictive swimming activity triggered a coupled rhythmic discharge in the left LR motor nerve and a correlated angular movement of the right eye, overall corresponding to a conjugate-like oculomotor activity (Figure 4B_i_). In this example, a leftward movement of the right eye presented a minimal response latency and a slight phase delay with the burst recorded in the left LR motor nerve (17.53 ± 1.84 ms and 40.97 ± 5.13°, respectively; Figure 4B_ii_). Those left LR bursts presented also comparable reduced latencies and discharged almost in phase with the right Vr-20 bursts (19.58 ± 2.09 ms and 48.28 ± 5.53°, respectively; Figure 4B_ii_). In contrast, the right Vr-5 bursts showed an important phase lead with the left LR bursts (263.54 ± 5.24° for a latency between those bursts of 37.51 ± 2.20 ms; Figure 4B_ii;_ see also Supplementary Figure S1). Statistical analysis based on different isolated *in vitro* preparations (Figure 4B_iii_) where spino-extraocular motor coupling was recorded between left LR, right Vr-5 and Vr-20 confirmed that LR bursts occurred with a small phase lead with the contralateral Vr-20 discharge (Figure 4B_iii_) but with a significant phase lag with the contralateral Vr-5 bursts (Figure 4B_iii;_ see also Supplementary Figure S1). The Vr-5-to-LR phase shift decreased significantly (Figure 4B_iv_, right panel; see also Supplementary Figure S1) with fictive swimming frequency whereas the Vr-5-to-LR latency remained constant at 37.11 ± 2.74 ms (Figure 4B_iv_, left curve; see also Supplementary Figure S1). This last result was confirmed by increasing artificially the frequency of the fictive swimming activity in some preparations where rostral spinal segments (1–10th) then mid-caudal spinal segments (11–22th) were perfused independently with a high K ^+^(6 mM) Ringer solution (not shown). In this condition, we observed the same linear relationship for the phase shift and the latency between LR and Vr-5 as described above (in Figure 4B).

**FIGURE 5:**
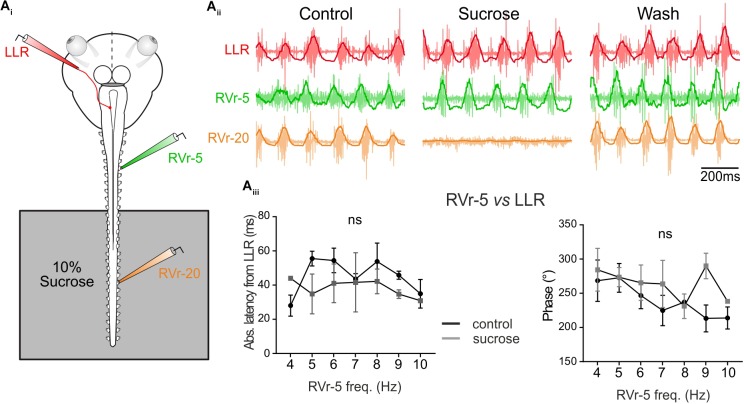
Temporal relationship of spino-extraocular motor coupling is only adjusted in rostral spinal CPG. **(A_i_)** Schematic of the *in vitro* preparation showing the recorded nerves and the experimental condition. (**A_ii_**-Control) Recording of spontaneous coordinated bursting discharge from left lateral rectus (LLR, red traces), and two contralateral spinal ventral roots (the 5th: RVr-5, green traces and the 20th: RVr-20 orange traces) during fictive swimming. Each raw trace was integrated and the result superimposed on the corresponding raw trace. Application of 10% sucrose on spinal segments more caudal than the 12th spinal ventral root (see schematic) blocked their activities as revealed by the Vr-20 recording without changing the temporal relationships between RVr-5 and LLR. **(A_iii_)** Absolute latency average between LLR and RVr-5 did not differ (two-way ANOVA, ns, *n* = 3) in control and in sucrose condition on a range of fictive swimming frequency (4–10 Hz). Consequently, for all swimming frequencies, the phase relationship between the RVr-5 and the LLR was not significantly different in control compared to sucrose condition.

The results presented in Figures 3, 4 show that locomotor-induced conjugate eye movements are produced by a spino-extraocular motor command elaborated by the rostral spinal CPGs (Figure 3; Lambert et al., 2012) but compensate (in the opposite direction) the undulatory movement of the mid-caudal part of the tail (Figure 4). The latency between the swimming locomotor bursts in rostral spinal segments (Vr-5) and the extraocular coupled motor signal (LR motor nerve) is maintained constant over the swimming frequency range (Figure 4B_iv_). This suggests that rostral CPGs, rather than mid-caudal CPGs, are responsible for tuning the temporal coupling between extraocular and spinal Vr motor signals. To confirm this hypothesis, the motor coupling between LR and Vr-5 motor nerves was investigated during blockade of swimming activity in mid-caudal spinal segments (Figure 5) by selectively bathing the spinal segments 11–22 (isolated with a Vaseline wall) in a Sucrose solution (Figure 5A_i_). This treatment abolished the fictive swimming activity in the mid-caudal locomotor networks (see the lack of bursting in Vr-20 in Figure 5A_ii_ under sucrose condition), but affect neither the fictive swimming activity in the rostral locomotor networks nor the spino-extraocular motor coupling (Figure 5A_ii_). The latency and phase shift (Figure 5A_iii_) between Vr-5 and LR motor nerve bursting discharge did not change significantly in sucrose condition, and this, whatever the swimming frequency (see Figure 4B_iv_). The fact that the temporal parameters (phase shift and latency) of the LR/Vr-5 motor coupling are kept constant despite the blockade of the mid-caudal locomotor networks confirms that the rostral locomotor networks adjust the timing of the spino-extraocular motor control in order to appropriately set the conjugated eye movements to compensate for the undulation of the mid-caudal part of the tail.

## Discussion

Spinal CPG efference copy has to be properly time calibrated to drive conjugate eye movements that ensure appropriate gaze stabilization during larval swimming. In this study, we showed that locomotor-induced oculomotor behavior is only expressed during propulsive swimming behavior, elicited by an intrinsic feed-forward efference copy signaling that originates from rostral spinal motor networks, as previously described *in vitro* (Lambert et al., 2012). Our results demonstrated that the feed-forward locomotor signal is timely set to generate a spino-extraocular motor coupling where the discharge bursts in extraocular motor nerves and in the contralateral 15–20th segmental spinal ventral roots are in phase during swimming episodes. This temporal tuning seems to be independent of the CPG rhythmic activity in spinal regions more distal than the 10th segment. In contrast to undulatory swimming in the lamprey where the spinal intersegmental delay varies with the swimming frequency (Wallen and Williams, 1984), in Xenopus, we observe that this delay is kept constant regardless of the swimming frequency ensuring that conjugate eye movements counteract the undulatory pattern of the mid-caudal section of the tail responsible for propulsive swimming behavior. Kinematic analysis (Wassersug and von Seckendorf Hoff, 1985; von Seckendorff Hoff and Wassersug, 1986) and computer modeling (Liu et al., 1996, 1997) of tail-based swimming have demonstrated that undulatory kinematic of larval amphibians differs from that of fish because of morphological differences and the fact that this kinematic is adapted to their particular developmental and metamorphosis constraints. Empiric studies based on high-speed video recordings of freely swimming larval frogs showed that the medial portion of the tail, where the fin is the largest, is the most efficient portion of the tail for generating thrust during forward swimming (Wassersug and von Seckendorf Hoff, 1985). Conversely the more caudal portion of the tail did not have a major role in thrust production but rather was involved in reducing turbulence around the larvae (Wassersug and von Seckendorf Hoff, 1985; Van Buskirk and McCollum, 2000). More recently, hydrodynamic model studies measured the maximum flow velocity vector and water pressure at the mid-tail portion, confirming the crucial role of this tail region in the production of thrust during tadpole swimming (Liu et al., 1996, 1997). In addition, Liu and colleagues revealed the existence of a non-propulsive ‘dead water zone’ just behind the head, at the most anterior part of the tail where hindlimbs grow. They hypothesized that this area of reduced flow velocity and pressure at the rostral part of the tail could provide a space where hindlimbs would develop without affecting the swimming-evoked water turbulence. This hypothesis is supported by behavioral observations of xenopus metamorphic stages (stage 58–60) where hindlimbs are maintained elongated along the body axis during undulatory swimming (Combes et al., 2004; von Uckermann et al., 2016).

Based on these kinematic studies we propose the following hypothesis to explain the temporal calibration of the locomotor-induced oculomotor behavior. Spino-extraocular pathways couple a propagated multi-segmental and rhythmic locomotor CPG activity with a simple alternated extraocular motor activity. To efficiently stabilize the gaze, locomotor-induced conjugate eye movements cannot simultaneously compensate all tail regions’ movements during swimming but must be time-settled to the mid-caudal tail region responsible for the power-stroke of swimming movements. By initiating the alternated myotomal contraction, the upper spinal segments (until 10th) determine the intersegmental rostro-caudal delay that set the timing of the undulatory swimming pattern. Therefore, those rostral swimming CPGs elicit ascending commands to extraocular motoneurons with a temporal calibration that produce conjugated eye movements counteracting specifically the undulation of the mid-caudal tail region, which is the most biomechanically efficient region to produce propulsive forward swimming.

One may wonder why the signals for spino-extraocular coupling arise principally from rhythmically active CPG networks in the rostral region and not directly from those innervating the myotomes of the middle part of the tail compensated by conjugate eye movements. We can notice that the rostral segments involved in the spino-ocular coordination are the only ones that remain after metamorphosis, when the tadpole changes into a frog and the tail disappears. Indeed, we have recently shown that the efferent copy is still present in the postmetamorphic animal, although adapted to the new mode of locomotion of the animal (von Uckermann et al., 2016). We can therefore imagine that the spinal-brainstem circuitry involved in locomotor-induced gaze stabilization is maintained throughout metamorphosis. The need to adapt locomotor-induced oculomotor behavior to the propulsive phase of the swimming pattern seems to be a general feature found in the amphibians’ lifespan. Indeed, in adult frogs the hydrodynamic thrust is produced by the synchronized extension of bilateral hindlimbs (Richards and Biewener, 2007; Richards, 2010; Richards and Clemente, 2013). Interestingly video recordings in adult xenopus as well as *in vitro* approaches showed compensatory eye movements in phase with the hindlimb extension phase (von Uckermann et al., 2013; Bacque-Cazenave et al., 2017).

Swimming kinematic parameters as well as spino-extraocular temporal parameters were comparable between all experimental conditions used to quantify the locomotor-driven oculomotor behavior and its electrophysiological correlates. Nonetheless some variability was observed between *in vitro* fictive swimming sequences and *in vivo* unrestrained swimming behavior. First, the frequency range was lower in fictive compared to free swimming conditions. Second, the phase shift between the LR motor nerve and rostral Vrs were reduced in isolated preparation compared to free swimming condition. Sensory feedback from the tail undulatory movement normally comprises spinal proprioceptive inputs from myotome contraction, lateral line neuromast inputs from water flow detection and central canal fluid movement detection from mechanosensory neurons. These sensory signals are known to ensure a multimodal control of locomotion (Haehnel-Taguchi et al., 2014; Knafo et al., 2017). In many other vertebrate and invertebrate models (Bucher et al., 2003; Pearson, 2008), sensory feedback ensure that the CPG activity is continuously shaped by the actual conditions to produce well-adapted and robust movements, therefore allowing a correct transition between locomotion phases. As a result, higher motor nerves discharge frequencies are observed in presence compared to in absence of sensory feedback (Chung et al., 2015). Therefore, both the lower swimming frequency and the related reduced LR/Vr-5 phase shift observed in isolated *in vitro* preparations could be due to the absence of sensory feedback inputs normally originating from tail undulation and its interaction with the aquatic environment. Indeed, proprioceptive and lateral line sensory signals might play a role in the adjustment of the temporal calibration and the strength of the spino-extraocular motor command in response to the swimming frequency variation.

## Conclusion

This study brought a quantified behavioral supply to our previous work focused on the understanding of the central pathways responsible for locomotor efference copy gaze stabilizing mechanism (Combes et al., 2008; Lambert et al., 2012). It sets the basis for understanding the mechanisms of locomotor-induced oculomotor behavior in larval frog during stereotypical movements such as turning, fast start or escape.

## Author Contributions

JB-C performed the experiments, acquired and analyzed the data, designed the figures, and wrote the manuscript. GC contributed to the software conception and testing for video tracking. MB conceived the idea and wrote the manuscript. FL conceived the idea, performed the experiments, acquired the data, designed the figures, and wrote the manuscript. DC conceived the idea and wrote the manuscript.

## Conflict of Interest Statement

The authors declare that the research was conducted in the absence of any commercial or financial relationships that could be construed as a potential conflict of interest.

## Footnotes

1 http://xenopus.univ-rennes1.fr/

## Supplementary Material

FIGURE S1, Supplemental data for Figure 4 Histograms and Polar plots showing the time relationships (latency and phase) between the 1st tail section (sct) and the Leye and 4th section in semi-intact preparations **(A)**; between the RVr-5 and Reye, LLR and RVr-20 **(B)** recorded *in vitro;* between RVr-5 and LLR or RVr-20 **(C)** and between RVr-5 and LLR **(D)** The four panels are, respectively, equivalent to A_ii_, B_ii_, B_iii_, and B_iv_ of Figure 4 but by taking either the 1st sct **(A)** or the RVr-5 **(B–D)** as the phase marker. **A**; Mann Whitney test, *p* < 0,01, *n* = 5. **B**; histogram statistics: Dunn’s multiple comparisons test, *p* < 0.01 and *p* < 0.0001; polar plot statistic: Watson–Williams *F*-test, *p* < 0.001; 61 cycles. **C**; histogram statistics: Mann–Whitney test, *p* < 0,05, *n* = 10 and *n* = 7; polar plot statistic: Watson–Williams *F*-test, *p* < 0.001. **D**; left side plot: Kruskal–Wallis test, ns, *n* = 8; right side plot: Watson–Williams *F*-test, *p* < 0.05, *n* = 8.
