# Supplementary figures and images for: Temporal Relationship of Ocular and Tail Segmental Movements Underlying Locomotor-Induced Gaze Stabilization During Undulatory Swimming in Larval Xenopus
Source: Front Neural Circuits. 2018 Oct 29;12:95. doi: 10.3389/fncir.2018.00095 (PMC6216112; doi:10.3389/fncir.2018.00095)

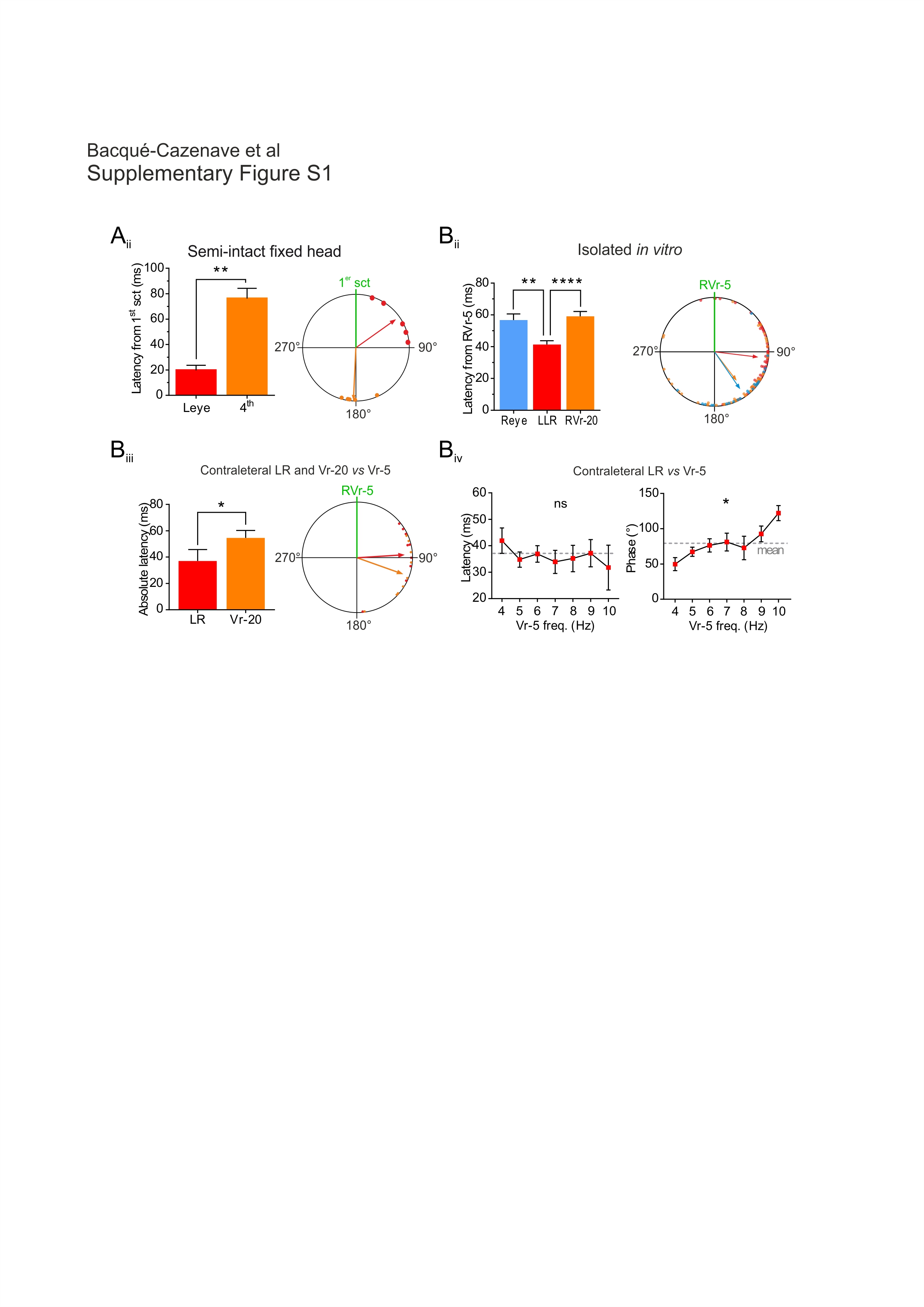

Supplement: FIGURE S1, Supplemental data for Figure 4 — Histograms and Polar plots showing the time relationships (latency and phase) between the 1st tail section (sct) and the Leye and 4th section in semi-intact preparations (A); between the RVr-5 and Reye, LLR and RVr-20 (B) recorded in vitro; between RVr-5 and LLR or RVr-20 (C) and between RVr-5 and LLR (D) The four panels are, respectively, equivalent to Aii, Bii, Biii, and Biv of Figure 4 but by taking either the 1st sct (A) or the RVr-5 (B–D) as the phase marker. A; Mann Whitney test, p < 0,01, n = 5. B; histogram statistics: Dunn’s multiple comparisons test, p < 0.01 and p < 0.0001; polar plot statistic: Watson–Williams F-test, p < 0.001; 61 cycles. C; histogram statistics: Mann–Whitney test, p < 0,05, n = 10 and n = 7; polar plot statistic: Watson–Williams F-test, p < 0.001. D; left side plot: Kruskal–Wallis test, ns, n = 8; right side plot: Watson–Williams F-test, p < 0.05, n = 8. [file Image_1.jpg]
