# Supplementary material for: Temporal Relationship of Ocular and Tail Segmental Movements Underlying Locomotor-Induced Gaze Stabilization During Undulatory Swimming in Larval Xenopus
Source: Front Neural Circuits. 2018 Oct 29;12:95. doi: 10.3389/fncir.2018.00095 (PMC6216112; doi:10.3389/fncir.2018.00095)
Supplement: Supplementary file 2 [file Data_Sheet_1.pdf]

Supplemental data source

Fig. 1D

Frequency (Hz)		
Free swim.	head fixed	*In vitro*
12,29 ± 0,84	7,20 ± 0,42	6,60 ± 0,36

Fig. 1E

Amplitude (°)	
head fixed	*In vitro*
22,77 ± 3,09	18,32 ± 3,56

Fig. $2A_{ii}$

Latency from 1st tail section (ms)			
2nd	4th	6th	7th
21,7 ± 1,75	44,11 ± 4,66	58,69 ± 7,91	69,05 ± 9,15

Fig. $2A_{ii}$

Phase relationship to 1st tail section (°)											
2nd			4th			6th			7th		
μ	r	p	μ	r	p	μ	r	p	μ	r	p
84,77	0,75	<0,01	180,23	0,7	<0,01	236,11	0,47	<0,05	287,82	0,41	>0,05

Fig. $2B_{ii}$

Latency from 1st tail section (ms)			
2nd	4th	6th	7th
37,91 ± 9,34	75,9 ± 6,56	97,78 ± 7,56	108,2 ± 9,58

Fig. $2B_{ii}$

Phase relationship to 1st tail section (°)											
2nd			4th			6th			7th		
μ	r	p	μ	r	p	μ	r	p	μ	r	p
105,67	0,58	<0,05	181,88	0,75	<0,001	242,54	0,75	<0,001	299,46	0,7	>0,01

Fig. $2C_{ii}$

Latency from Vr-5 (ms)		
Vr-10	Vr-15	Vr-20
16,95 ± 1,00	37,57 ± 1,11	58,41 ± 1,27

Fig. $2C_{ii}$

Phase relationship to Vr-5 (°)								
Vr-10			Vr-15			Vr-20		
μ	r	p	μ	r	p	μ	r	p
36,91	0,92	<0,0001	81,66	0,92	<0,0001	129,19	0,89	<0,0001

Fig. $2C_{iii}$

Latency from Vr-5 (ms)						
4Hz	5Hz	6HZ	7Hz	8Hz	9Hz	10Hz
52,07 ± 10,38	49.27 ± 6,75	51.18 ± 5.30	52,43 ± 5.27	56,01 ± 6,55	61,14 ± 7,95	54,4 ± 9,09

Fig. $2C_{iii}$

Phase relationship to Vr-5 (°)																				
4Hz			5Hz			6Hz			7Hz			8Hz			9Hz			10Hz		
μ	r	p	μ	r	p	μ	r	p	μ	r	p	μ	r	p	μ	r	p	μ	r	p
83,15	0,9	<0,01	91,52	0,92	<0,01	112,93	0,86	<0,01	128,8	0,88	<0,01	144,04	0,9	<0,01	151,14	0,85	<0,05	161,01	0,95	<0,05

Fig. $3B_{ii}$

Linear regression					
1st sct		4th sct		4th sct no swim	
r^2	s	r^2	s	r^2	s
0,46	0,51	0,21	0,47	0,02	-0,02

Fig. $3C_{ii}$

Linear regression			
Vr-5		Vr-20	
r^2	s	r^2	s
0,8	0,83	0,18	0,11

Fig. $4A_{ii}$

Latency from Leye	
1st sct	4th sct
19,71 ± 3,98	50,14 ± 9,15

Fig. $4A_{ii}$

Phase relationship to Leye (°)					
1st			4th		
μ	r	p	μ	r	p
305,25	0,908	<0,01	130,7	0,741	≤0,05

Fig. 4B$_{ii}$

Latency from Leye		
Reye	RVr-5	RVr-20
17,53 ± 1,84	37,51 ± 2,20	19,58 ± 2,09

Fig. 4B$_{ii}$

Phase relationship to LLR (°)								
Reye			1st			4th		
µ	r	p	µ	r	p	µ	r	p
40,98	0,9	<0,0001	263,53	0,791	<0,0001	48,284	0,77	<0,0001

Fig. 4B$_{iii}$

Latency from LLR	
RVr-5	RVr-20
37,11 ± 2,74	12,93 ± 4,31

Fig. 4B$_{iii}$

Phase relationship to LLR (°)					
Vr-5			Vr-20		
µ	r	p	µ	r	p
22,08	0,689	<0,01	274,41	0,65	<0,001

Fig. 4B$_{iv}$

Latency from LLR (ms)						
4Hz	5Hz	6HZ	7Hz	8Hz	9Hz	10Hz
41,92 ± 4,81	34,80 ± 2,88	36,91 ± 3,13	33,91 ± 4,36	35,20 ± 4,98	37,16 ± 5,16	31,76 ± 8,45

Fig. 4B$_{iv}$

Phase relationship to LLR (°)																				
4Hz			5Hz			6Hz			7Hz			8Hz			9Hz			10Hz		
µ	r	p	µ	r	p	µ	r	p	µ	r	p	µ	r	p	µ	r	p	µ	r	p
312,45	0,94	<0,001	292,12	0,96	<0,0001	281,88	0,93	<0,0001	278,9	0,9	<0,001	280,26	0,82	<0,01	267,32	0,92	<0,01	237,78	0,98	ns

Supplementary Figure S1

A$_{ii}$

Latency from 1st sct	
Leye	4th sct
19,71 ± 3,98	76,24 ± 8,03

A$_{ii}$

Phase relationship to 1st sct (°)					
Leye			4th		
µ	r	p	µ	r	p
54,22	0,91	<0,01	182,59	0,97	<0,01

B$_{ii}$

Latency from RVr-5		
Reye	LLR	RVr-20
56,78 ± 3,88	41,47 ± 2,31	59,18 ± 3,09

B$_{ii}$

Phase relationship to RVr-5 (°)								
Reye			LLR			RVr-20		
µ	r	p	µ	r	p	µ	r	p
131,32	0,823	<0,0001	96,86	0,787	<0,0001	142,4	0,64	<0,0001

B$_{iii}$

Latency from Vr-5	
LR	Vr-20
37,11 ± 2,74	54,56 ± 5,77

B$_{iii}$

Phase relationship to Vr-5 (°)					
LR			Vr-20		
µ	r	p	µ	r	p
85,59	0,65	<0,001	109,3	0,67	<0,001

B$_{iv}$

Latency from RVr-5 (ms)						
4Hz	5Hz	6HZ	7Hz	8Hz	9Hz	10Hz
41,92 ± 4,81	34,80 ± 2,88	36,91 ± 3,13	33,91 ± 4,36	35,20 ± 4,98	37,16 ± 5,16	31,76 ± 8,45

B$_{iv}$

Phase relationship to LLR (°)																				
4Hz			5Hz			6Hz			7Hz			8Hz			9Hz			10Hz		
µ	r	p	µ	r	p	µ	r	p	µ	r	p	µ	r	p	µ	r	p	µ	r	p
49,8	0,94	<0,001	67,32	0,96	<0,0001	76,37	0,93	<0,0001	81,12	0,9	<0,001	72,77	0,82	<0,01	92,59	0,92	<0,01	122,2	0,98	ns

Fig. 5A$_{iii}$

	Latency from LLR (ms)						
	4Hz	5Hz	6HZ	7Hz	8Hz	9Hz	10Hz
control	28,05 ± 6,13	55,51 ± 4,31	54,46 ± 7,17	43,59 ± 3,08	53,83 ± 10,75	45,82 ± 2,33	34,94 ± 8,38
sucrose	43,96 ± 0,00	34,90 ± 11,65	41,06 ± 11,5	41,67 ± 17,31	42,22 ± 7,27	34,78 ± 2,50	30,96 ± 0,00

	Phase relationship to LLR (°)						
	4Hz	5Hz	6HZ	7Hz	8Hz	9Hz	10Hz
control	268,5 ± 30,1	272,6 ± 21,1	246,7 ± 19,3	224,9 ± 22,2	237,1 ± 1,7	213,3 ± 19,6	213,9 ± 16,1
sucrose	284,4 ± 31,3	273,7 ± 14,1	265,5 ± 25,8	263,8 ± 34,5	231,1 ± 17,9	290,0 ± 18,7	283,3 ± 0,00
